# Methylomic predictors demonstrate the role of NF-κB in old-age mortality and are unrelated to the aging-associated epigenetic drift

**DOI:** 10.18632/oncotarget.8278

**Published:** 2016-03-22

**Authors:** Juulia Jylhävä, Laura Kananen, Jani Raitanen, Saara Marttila, Tapio Nevalainen, Antti Hervonen, Marja Jylhä, Mikko Hurme

**Affiliations:** ^1^ Department of Microbiology and Immunology, School of Medicine, University of Tampere, Tampere, Finland; ^2^ Gerontology Research Center, University of Tampere, Tampere, Finland; ^3^ School of Health Sciences, University of Tampere, Tampere, Finland; ^4^ UKK Institute for Health Promotion Research, Tampere, Finland; ^5^ Fimlab Laboratories, Tampere, Finland

**Keywords:** methylation, mortality, aging, longevity, Cox model, Gerotarget

## Abstract

Changes in the DNA methylation (DNAm) landscape have been implicated in aging and cellular senescence. To unravel the role of specific DNAm patterns in late-life survival, we performed genome-wide methylation profiling in nonagenarians (*n*=111) and determined the performance of the methylomic predictors and conventional risk markers in a longitudinal setting. The survival model containing only the methylomic markers was superior in terms of predictive accuracy compared with the model containing only the conventional predictors or the model containing conventional predictors combined with the methylomic markers. At the 2.55-year follow-up, we identified 19 mortality-associated (false-discovery rate <0.5) CpG sites that mapped to genes functionally clustering around the nuclear factor kappa B (NF-κB) complex. Interestingly, none of the mortality-associated CpG sites overlapped with the established aging-associated DNAm sites. Our results are in line with previous findings on the role of NF-κB in controlling animal life spans and demonstrate the role of this complex in human longevity.

## INTRODUCTION

The influential role of genomic factors, such as DNA methylation (DNAm) in the course of development, aging and age-related pathologies is well established. Several studies have also reproducibly demonstrated that the level of methylation at specific CpG sites changes as a function of age [1-5], hence providing a marker of chronological and, potentially, biological age. An intriguing characteristic of age-related DNAm signatures is that many of the age-associated DNAm changes have been observed to be common in several different tissues, such as whole blood, brain, lung and cervix [1, 3, 6]. These observations suggest that a global mechanism(s) might be responsible for age-associated modifications in the epigenetic landscape. Nevertheless, studies with monozygotic twins have demonstrated that the rate of divergence in methylomic patterns increases with age [7, 8], suggesting that the age-related modifications in DNAm are also subject to various environmental, stochastic and life style-related effects.

However, the consequences of the aging-accompanied DNAm alterations for late-life health and functional abilities are largely unknown. A recent epigenome-wide association study (EWAS) demonstrated that the association between age-related DNAm changes and healthy aging phenotypes in individuals 32-80 years of age is negligible [8]. The results of this study also reveal that the DNAm regions associated with aging phenotypes are distinct from those associated with chronological age. These findings suggest that the CpG sites involved in health-related outcomes in later life are largely regulated by sites other than the established age-related DNAm regions [8]. In addition, using an EWAS approach, we have recently demonstrated that the CpG sites that are associated with aging-related inflammation, i.e., inflammaging [9] are largely different from the sites associated with age [5]. This phenomenon is also observable in regard to gene expression profiles and old age mortality. We have previously demonstrated that the genes exhibiting aging-related changes in expression levels are predominantly different from those that predict mortality in late life [10]. These findings underscore the complexity and unknown nature of the genomic factors that control the human health span and late-life events.

Nevertheless, the mortality-predicting genes in our previous study were found to be functionally connected to the nuclear factor kappa B (NF-κB) complex, which is a central mediator in immunoinflammatory responses and has been advocated as the culprit in aging and cellular senescence (reviewed in [11]). Aberrant activation of NF-κB has been reported in various age-associated conditions, such as neurodegeneration, immunosenescence, inflammaging, sarcopenia and osteoporosis (reviewed in [12-14]), whereas studies involving mouse models have observed that NF-κB activation is a key determinant of accelerated aging and longevity [15, 16]. In the mouse models, it was demonstrated that the hypothalamic activation of NF-κB is a driving force of systemic aging through immune-endocrine connections [16].

Life span regulation in humans is a multifactorial process, and very little is known about the genomic determinants that control late-life mortality after the ages of the common killers, i.e., cardiovascular events and cancer, have passed. In this study, we sought to explore how the human genome-wide methylome is associated with old-age survival within a shorter (2.55 years) and a longer (4 years) follow-up time. A large panel of traditional (bio)markers and mortality risk factors was assessed alongside the methylomic markers to elucidate the relationship between the aging-related biophysiological changes and epigenetics.

## RESULTS

The characteristics of the study population and distribution of the variables in the population with methylation data available (*n* = 111) are presented in Table 1. The variables (i.e., the conventional markers) exhibiting significant (*p* < 0.05) univariate and multivariate associations at the 2.55 follow-up are presented in Supplementary Table 1. The predictors remaining in the multivariate model, body mass index (BMI) and Mini-Mental State Examination (MMSE) test score, were used as the model factors in the assessment of the predictive accuracy of modeling (see Methods). The measure of “epigenetic clock” [17], the DNA methylation age was not predictive of mortality in our cohort (*p* = 0.733).

**Table 1:** Characteristics of the study population (*n* = 111) Distributions of the variables are presented according to the data at the 2.55-years mortality follow-up.

	Non-survivors		Survivors	
Variable	Mean/Med	SEM/IQR/%	Mean/Med	SEM/IQR/%
Women (n/%)	27	75.0	54	72.0
Age (months)	1079.5	0.61	1080.2	0.37
Systolic blood pressure (mmHg)	145	4.6	149	3.4
Diastolic blood pressure (mmHg)*	71.5	13.5	74.0	19.0
Weight (kg)	61.9	2.2	70.6	1.6
BMI (kg/m*)	24.3	0.75	27.5	0.54
Waist circumference (cm)	89.6	2.1	95.5	1.4
Hip circumference (cm)*	98	10.0	102	12.0
MMSE*	23.5	8.0	26.0	4.0
Barthel index*	95.0	20.0	95	5.0
Handgrip (kg)*	18.0	11.0	20.0	7.0
Able to perform chair-rise test (***n*** = yes/%)	19	57.6	59	78.7
Able to perform chair-stand test (***n*** = yes/%)	22	71.0	62	82.7
Frailty index (n/%)				
Non-frail	3	8.3	26	34.7
Pre-frail	22	61.1	37	49.3
Frail	11	30.6	12	16.0
CRP level (ng/ml)*	1.8	3.3	1.9	3.5
IL-1β level (pg/ml)*	14.2	27.6	19.0	34.0
IL-6 level (pg/ml)*	4.5	3.3	3.8	3.8
IL-7 level (pg/ml)*	7.8	5.3	6.4	5.2
IL-10 level (pg/ml)*	1.8	1.5	1.5	2.6
cf-DNA level (μg/ml)*	0.93	0.19	0.87	0.16
Unmethylated cf-DNA level (μg/ml)*	0.75	0.20	0.67	0.15
Plasma mtDNA (copy number)*	4.30E*	2.37E*	3.75E*	2.09E*
*Alu* repeat cf-DNA (GE)*	74.4	50.4	66.8	38.3
DHEAS (μg/ml)*	0.25	0.48	0.25	0.31
Cortisol (ng/ml)*	133	54.3	117	68.0
IDO activity (Kyn/Trp)*	44.3	25.5	51.8	25.3
Anti-CMV antibody titer*	19.000	8.000	19.000	9000
Anti-EBV antibody titer*	405	315	410	410
DNAm age	76.1	1.04	76.1	0.64
CD3+ cells (%)*^a^	62.0	15.8	57.9	12.0
CD4+ cells (%)^b^	62.9	2.5	63.8	1.6
CD8+ cells (%)^b^	30.6	2.3	28.9	1.5
CD4+/CD8+ cells (ratio)*	2.4	2.3	2.3	2.4
CD4+CD28− cells (%)*	9.2	16.2	9.2	13.0
CD8+CD28− cells (%)	63.3	2.8	63.3	2.1
CD14+ cells (%)*^a^	8.3	5.9	8.1	6.3

* median values and IQR presented

a percentage of live-gated cells

b percentage of total T lymphocytes (CD3+ cells)

c percentage of CD4+ cells

d percentage of CD8+ cells

Abbreviations: BMI, body mass index; CD, cluster of differentiation; CMV, cytomegalovirus; CRP, C-reactive protein; cf-DNA, cell-free DNA; DHEAS, dehydroepiandrosterone sulfate; DNAm, DNA methylation; EBV, Epstein-Barr virus; GE, genomic equivalent; IDO, indoleamine 2,3-dioxygenase; IL, interleukin; Kyn, kynurenine; MMSE, Mini-Mental State Examination; mtDNA, mitochondrial DNA; Trp, tryptophan

**Figure 1:**
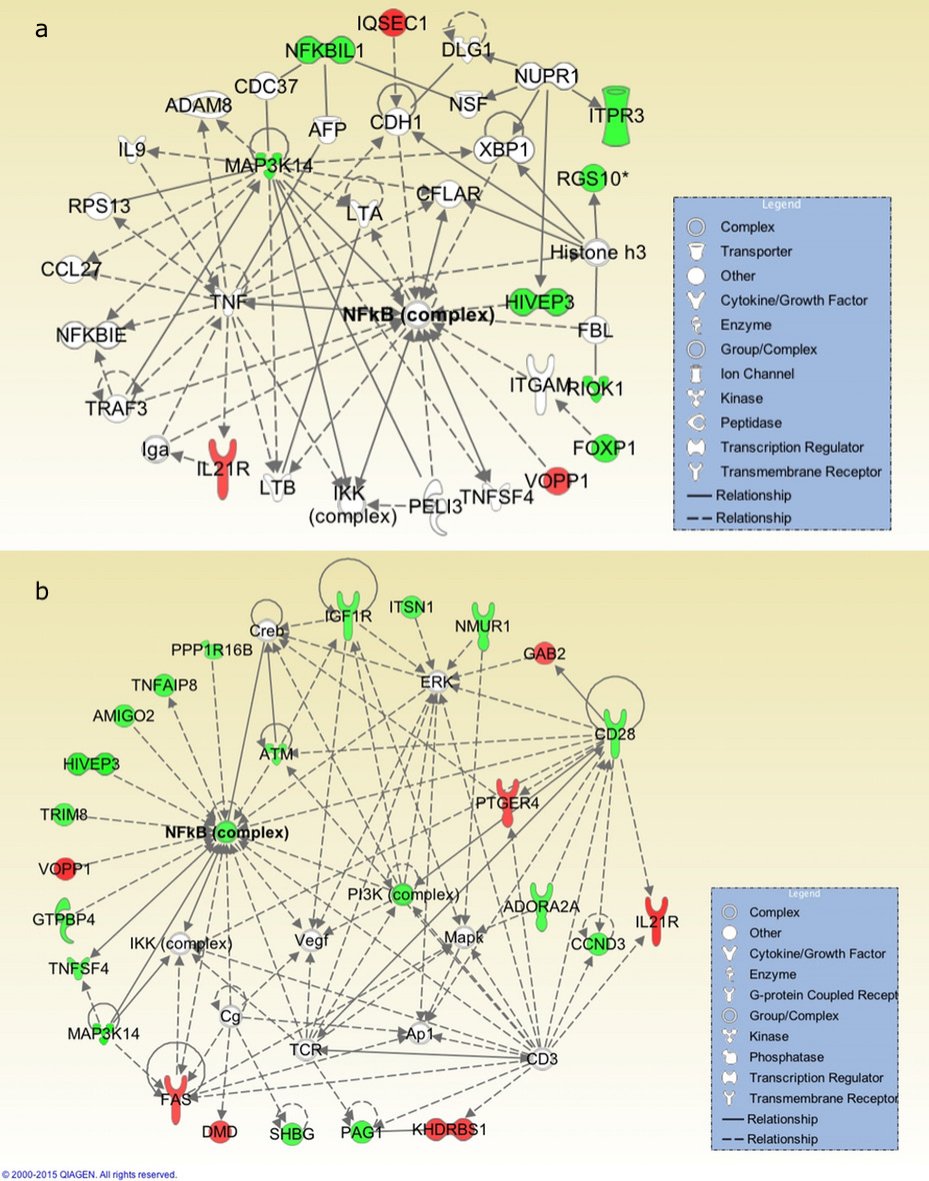
The highest-ranking networks from the 16 known genes harboring the top 19 significant (FDR < 0.5) CpG sites **a**. and from the genes harboring the top 250 CpG sites **b.** in the 2.55-year follow-up (*n* = 111). Both networks displayed NF-κB as a central node and were enriched for the common term *Hematological System Development and Function*. The green color of the molecule indicates that hypomethylation of a CpG site in the gene was associated with increased mortality, and the red color indicates that hypermethylation of a CpG site in the gene was associated with increased mortality. The networks were generated through the use of QIAGEN's Ingenuity Pathway Analysis (IPA^®^, QIAGEN Redwood City, www.qiagen.com/ingenuity).

In the Cox univariate assessment, 19,621 and 15,505 CpG sites were associated with mortality (*p* < 0.05) in the 2.55-year and 4-year follow-up data, respectively (Supplementary Tables 2 and 3). After B-H correction (FDR < 0.5), 19 CpG sites remained significant for the 2.55-year follow-up and 7 CpG sites for the 4-year follow-up data (Supplementary Tables 2 and 3). The Ingenuity Pathway Analysis (IPA) -generated network from the 16 known genes harboring the 19 significant CpG sites at the 2.55-year follow-up is presented in Figure 1a. This network displayed NF-κB as a central node and involved 10 of 16 of the genes mapped to the 19 mortality-associated CpG sites (FDR < 0.5). We also ran the IPA network and pathway analyses from the genes harboring the 250 top-ranking CpG sites according to the 2.55-year follow-up data (sites presented in Supplementary Table 2). The highest-ranking network in this analysis also placed NF-κB as a central complex (Figure 1b). The significant B-H-corrected canonical pathways from this data set are presented in Table 2. At the 4-year follow-up, the functional implications of the methylomic predictors were attenuated as no significant B-H -corrected canonical pathways were identified in IPA from the genes harboring the 250 highest-ranking CpG sites and no significant network enrichment was observed among the genes harboring the 7 CpG sites (FDR < 0.5).

Assessment of the predictive accuracy of the tested models revealed that the Ridge regression containing only the methylomic markers (Ridge1) performed better than the other models; i.e., a model containing only the conventional predictors, a Ridge regression model containing both the conventional predictors (Ridge2) and the methylomic markers and a model containing only the methylomic markers selected on the basis of their significance level in Cox univariate assessment. Specifically, the methylomic markers alone exhibited the smallest median deviance from the null model (Supplementary Figure 1), and were thus used in assessing the final mortality-predicting signature in the Cox multivariate model for 2.55-year follow-up data. The deviances of the conventional markers exhibited clearly the smallest variation but their median was nevertheless higher than that of the methylomic markers in Ridge1.

**Table 2:** Canonical pathways constructed from the genes harboring the top 250 CpG sites associated with mortality at the 2.55-year follow-up

Ingenuity Canonical Pathways	−log(p)*	Ratio	Molecules
Chronic Myeloid Leukemia Signaling	1.91	7.61E-02	*TGFBR2, GAB2, HDAC4, SMAD3, PIK3R2, NFKB1, ATM*
Germ Cell-Sertoli Cell Junction Signaling	1.58	5.13E-02	*TGFBR2, MAP3K14, MAP3K10, ACTA2, KEAP1, ITGA2, PIK3R2, ATM*
Role of NFAT in Cardiac Hypertrophy	1.58	4.55E-02	*IL6ST, TGFBR2, HDAC4, ITPR3, IGF1R, SLC8A3, PIK3R2, ATM*
Cell Cycle: G1/S Checkpoint Regulation	1.58	7,94E-02	*CCND2, HDAC4, CCND3, SMAD3, ATM*
Regulation of the Epithelial-Mesenchymal Transition Pathway	1.58	4.40E-02	*MAML1, TGFBR2, FZD3, SMAD3, PIK3R2, NFKB1, SMURF1, ATM*
iCOS-iCOSL Signaling in T Helper Cells	1.58	5.83E-02	*GAB2, CD28, ITPR3, PIK3R2, NFKB1, ATM*
Rac Signaling	1.58	5,83E-02	*CYFIP2, ITGA2, PIK3R2, NFKB1, ATM, ANK1*
NF-κB Activation by Viruses	1.58	6.85E-02	*MAP3K14, ITGA2, PIK3R2, NFKB1, ATM*
Hepatic Fibrosis/Hepatic Stellate Cell Activation	1.58	4.08E-02	*TGFBR2, TNFSF4, ACTA2, MYH14, SMAD3, IGF1R, NFKB1, FAS*
GADD45 Signaling	1.58	1.58E-01	*CCND2, CCND3, ATM*
PKCθ Signaling in T Lymphocytes	1.57	5.31E-02	*MAP3K14, MAP3K10, CD28, PIK3R2, NFKB1, ATM*
Molecular Mechanisms of Cancer	1.57	3.06E-02	*TGFBR2, GAB2, CCND2, CCND3, FZD3, SMAD3, ITGA2, PIK3R2, NFKB1, FAS, ATM*
CNTF Signaling	1.46	8.16E-02	*IL6ST, CNTF, PIK3R2, ATM*
B Cell Receptor Signaling	1.44	4.09E-02	*GAB2, MAP3K14, MAP3K10, PAG1, PIK3R2, NFKB1, ATM*
RANK Signaling in Osteoclasts	1.44	5.81E-02	*MAP3K14, MAP3K10, PIK3R2, NFKB1, ATM*
Virus Entry via Endocytic Pathways	1.44	5.62E-02	*ITSN1, ACTA2, ITGA2, PIK3R2, ATM*
Crosstalk between Dendritic Cells and Natural Killer Cells	1.44	5.62E-02	*CD28, ACTA2, KLRD1, NFKB1, FAS*
Lymphotoxin β Receptor Signaling	1.44	7.41E-02	*MAP3K14, PIK3R2, NFKB1, ATM*
Death Receptor Signaling	1.44	5.49E-02	*MAP3K14, ACTA2, PARP12, NFKB1, FAS*
Colorectal Cancer Metastasis Signaling	1.43	3.46E-02	*IL6ST, TGFBR2, FZD3, SMAD3, PIK3R2, NFKB1, PTGER4, ATM*
Myc Mediated Apoptosis Signaling	1.40	6.90E-02	*IGF1R, PIK3R2, FAS, ATM*
T Cell Receptor Signaling	1.40	5,21E-02	*CD28, PAG1, PIK3R2, NFKB1, ATM*
Estrogen-Dependent Breast Cancer Signaling	1.30	6.45E-02	*IGF1R, PIK3R2, NFKB1, ATM*
CD40 Signaling	1.30	6.25E-02	*MAP3K14, PIK3R2, NFKB1, ATM*
HGF Signaling	1.30	4.81E-02	*MAP3K14, MAP3K10, ITGA2, PIK3R2, ATM*
Pancreatic Adenocarcinoma Signaling	1.30	4.72E-02	*TGFBR2, SMAD3, PIK3R2, NFKB1, ATM*
NGF Signaling	1.30	4.72E-02	*MAP3K14, MAP3K10, PIK3R2, NFKB1, ATM*
T Helper Cell Differentiation	1.30	5.97E-02	*IL6ST, TGFBR2, CD28, IL21R*
IL-9 Signaling	1.30	8.82E-02	*PIK3R2, NFKB1, ATM*

* Benjamini-Hochberg-corrected *p*-value

The Ridge regression-organized 19 methylomic markers entered to the Cox multivariate model are presented in Supplementary Table 4. Inclusion of the methylomic markers in the final model was based on selection of the model with the best goodness of fit (Akaike Information criterion, AIC), which for the selected model was 239.0. The final Cox multivariate model is presented in Table 3 and the distributions of the beta values for the seven CpG sites (batch effect -corrected) included this mortality-predicting signature are presented in Supplementary Figure 2.

**Table 3:** The final mortality-predicting signature at the 2.55-year follow-up assessed from the Ridge regression -organized methylomic markers

	HR (95% CI)	S.E.	Z	*p*
cg08421934 (*NA*)	0.41 (0.26-0.64)	0.10	−3.84	<0.001
cg15770702 (*MAP3K14*)	0.40 (0.27-0.61)	0.08	−4.38	<0.001
cg08596308 (A*TP6V1G2*; *NFKBIL1*)	0.50 (0.34-0.73)	0.10	−3.60	<0.001
cg23282964 (*RIOK1*)	0.56 (0.37-0.84)	0.12	−2.82	0.005
cg16720947 (*PLEC1*)	0.52 (0.34-0.80)	0.13	−2.94	0.003
cg27027151 (*IL21R*)	2.09 (1.44-3.02)	0.39	3.90	<0.001
cg26843567 (*NA*)	0.68 (0.46-0.99)	0.13	−2.01	0.045

Abbreviations: CI, confidence interval; HR, hazard ratio; NA, not available; S.E., standard error

The discriminative power (Harrell's *C*) for this model was 89.9%. The proportionality assumption in the Cox Regression model was tested using the global test by calculating the scaled Schoenfeld residuals for each of the independent predictors in the final Cox model. Statistically significant dependence of mortality on time was not observed (*p* = 0.280) indicating that the proportionality assumption was not violated.

Due to the small number of mortality-associated CpG sites in the methylomic data at the 4-year follow-up, no comparison of the prediction accuracies of the different modeling options or assessment of the final mortality-predicting signature was performed for the 4-year mortality data.

Correlation analysis between the methylation levels in the mortality-associated CpG sites and the corresponding gene product(s) revealed a significant correlation between three CpG site/transcript pairs. Inverse correlations were observed between the cg03348466 (*CRTC3*) and CRTC3 mRNA level and between cg04182483 (*RGS10*) and the RGS10 mRNA level. A direct correlation was observed between cg22794214 (*HIVEP3*) and HIVEP3 mRNA level. All the correlations are presented in Supplementary Table 5.

Analysis of the genomic locations of the top 19 CpG sites (FDR < 0.5, in Supplementary Table 2) for transcription factor (TF) binding sites and other genomic regulatory elements revealed that a majority of the sites were located on active *cis*-regulatory regions; they either harbored TF binding sites, DNAse I hypersensitivity regions, and/or were identified as “Predicted promoter region including transcription start site (TSS)”, “Predicted enhancer (E)” or “Predicted weak enhancer or open chromatin (WE)”. In addition, six CpG sites demonstrated functional significance as they were annotated for “Predicted transcribed region (T)”. The most abundant TFs were POLR2A and RELA which both had binding sites on four CpG site loci. Full data of this assessment are presented in Table 4.

**Table 4:** Assessment of the 19 mortality associated CpG sites (FDR<0.5) in the 2.55-year follow-up (*n* = 111) for transcription factor binding sites and other functional genomic elements using ENCODE data in the UCSC genome browser

CpG site (gene)	GRCh37/hg19 coordinate	Transcription Factors	Genome status	DNAse I Hypersensitivity Cluster
cg24859528 *(IQSEC1)*	chr3:12941421		T	NO
cg03348466 *(CRTC3)*	chr15:91104770	CEBPB	T	YES
cg02395768 *(ATP5SL)*	chr19:41945578	SIN3AK20, POLR2A, SP2, SP1, CHD2, NFYB, PBX3, MAZ, NFIC, GTF2F1, MTA3, TAF1, TBL1XR1, JUND, KDM5B, STAT5A, HDAC1, SAP30, FOS, YY1, PHF8, FOXM1, TBP, CEBPB, REST, TCF12, IRF1, TEAD4, ZBTB7A, GABPA, MEF2A, PML, RELA	TSS	YES
cg15770702 *(MAP3K14*)	chr17:43384845	PML, STAT5A, NFATC1, CEBPB, BCL3, TCF12, EBF1, FOXM1, EP300, RELA, STAT3, NFIC, TBL1XR1, JUND, MEF2A, PAX5, BHLHE40, MEF2C, ATF2, SP1, BATF, RUNX3, IRF4, BCL11A	TSS/T	YES
cg16720947 *(PLEC1*)	chr8:145048137		n.a.	YES
cg22794214 *(HIVEP3)*	chr1:42123463	CTCF	WE/R	YES
cg08596308 *(ATP6V1G2; NFKBIL1)*	chr6:31516045	CHD1, RBBP5, ZNF274, POLR2A, E2F6, E2F4, KDM5B, MYC, MAX, MAZ	TSS	YES
cg23282964 *(RIOK1)*	chr6:7417780		T	NO
cg21200667 (NA)	chr2:30628085		R	YES
cg08421934 (NA)	chr6:33942413		R	NO
cg08486432 *(ITPR3)*	chr6:33598003		T/R	YES
cg08352439 *(VOPP1)*	chr7:55637123	POLR2A, POU2F2	TSS	YES
cg25356639 *(FOXP1)*	chr3:71349304		R	NO
cg04395703 *(METAP1)*	chr4:99982762		T	YES
cg03171419 *(GPR124*)	chr8:37700802	POLR2A	T	YES
cg26843567 (NA)	chr12:104846281		R	YES
cg00291478 *(RGS10)*	chr10:121301041	RELA, RUNX3, RBBP5	TSS	YES
cg27027151 *(IL21R)*	chr16:27461638	POLR2A, MTA3, NFATC1, RELA, BCLAF1, EBF1	E/R	YES
cg04182483 *(RGS10)*	chr10:121259610		T	NO

Abbreviations: TSS, Predicted promoter region including transcription start site (TSS); T, Predicted transcribed region; WE, Predicted weak enhancer or open chromatin cis-regulatory element; E, Predicted enhancer; R, Predicted Repressed or Low Activity region

## DISCUSSION

We have previously demonstrated, using genome-wide gene expression data, that the NF-κB complex is centrally involved in controlling human old-age mortality [10]. In the present study, we expanded the examination of the genomic factors regulating late-life survival by analyzing the predictive ability of genome-wide methylomic data at the 2.55-year follow-up. The results of this study corroborate the role of NF-κB in all-cause elderly mortality; the molecular network constructed from the genes harboring the mortality-associated CpG sites displayed the NF-κB complex as a central mediator (Figure 1). The genes *nuclear factor of kappa light polypeptide gene enhancer in B-cells 1* (*NFKB1*) and *ataxia telangiectasia mutated* (*ATM*) were also identified in the network. Intriguingly, both NFKB1 and ATM have previously been linked with accelerated aging and cellular senescence in studies with genetically engineered mice [15, 18, 19]. These studies advocated that NFKB1 and ATM*-*regulated aberrant NF-κB activation and the ensuing chronic systemic inflammatory state are the ultimate drivers of senescence and aging-associated deterioration [15, 18]. Although our data do not provide a mechanistic link between the hypomethylation of these CpG sites and the risk of mortality, we speculate that the mechanism involves an inflammatory component by which the genomic factors control late-life mortality.

Analysis of the 19 mortality-associated CpG sites (FDR < 0.5) for genomic regulatory elements revealed that a majority of the sites were located on active *cis*-regulatory regions (Table 4). That is, they harbored TF binding sites, located on DNAse I hypersensitivity areas and/or displayed one of the following predicted genomic states: promoter region including transcription start site, enhancer or weak enhancer/open chromatin. It is possible that the association between these sites and longevity is mediated through altered binding of TFs or methyl-binding domain proteins, of which the latter recruit chromatin-modifying proteins to achieve a repressive chromatin state. However, our data do not allow us to determine whether disrupted regulation of chromatin permissiveness underlies the increased mortality risk. Interestingly, RELA, which is a subunit of the NF- κB complex, was identified to have a binding site on four of the analyzed 19 CpG sites. This observation further supports the hypothesis of the functional role of NF-κB in old-age mortality.

Region of a predicted transcription start site was observed for cg02395768 *(ATP5SL),* cg15770702 *(MAP3K14*), cg08596308 *(ATP6V1G2; NFKBIL1)*, cg08352439 *(VOPP1)* and cg00291478 *(RGS10).* However, the methylation levels in these sites were not correlated with gene expression (Supplementary Table 5). Instead, methylation levels of cg03348466 (*CRTC3*), cg22794214 (*HIVEP3*) and cg04182483 (*RGS10*) correlated with the corresponding transcript expression level. The observation that the correlations were overall modest is, however, in line with previous findings on minimal correlations between age-associated changes methylation and transcription [5, 20, 21]. Six sites, including cg03348466 (*CRTC3*) and cg04182483 (*RGS10*) resided in predicted transcribed area, and can hence also be considered functionally significant. The potential regulatory role of these sites (in the gene body region) may involve e.g. alternative splicing. However, the exact mechanism connecting the mortality-associated changes in methylation to alternative splicing requires further research.

The canonical pathways constructed from the genes harboring the top 250 mortality-associated CpG sites at the 2.55-year follow-up covered a wide variety of cellular signaling functions among which several inflammation and immunity-related processes were represented. Interestingly, pathways termed NF-κB Activation by Viruses, GADD45 Signaling and Cell Cycle: G1/S Checkpoint Regulation were also identified. The emergence of these pathways suggests that NF-κB might also be involved late-life control of cellular growth and survival, DNA repair and apoptosis, as these functions are ascribed to the induction of the NF-κB- GADD45 cascade [22]. Interestingly, in our previous paper on the transcriptomic mortality predictors, we observed that an increased expression of *GADD45B* was predictive of an increased risk of mortality in these nonagenarians [10].

However, as the number of mortality-associated CpG sites was markedly reduced from the 2.55-years follow-up to the 4-years follow-up, we speculate that the methylomic markers might exhibit a dynamic nature even in the extreme ages. That is, a substantial part of the genomic CpG sites might be constantly remodeled, and during 4 years, their methylation levels are likely to change to an extent that their predictive ability in our population is reduced. The longer follow-up time also allows more time for stochastic mortality determinants, such as trauma, to operate, which may thus weaken the role of the genomic predictors.

Although the methylomic markers did not exhibit very strong statistical significances after FDR-correction and we used a liberal threshold for including them in the Ridge regression (FDR < 0.5), the methylomic data demonstrated good performance in terms of generalizability and discriminative power. Specifically, the methylomic data alone exhibited better predictive accuracy than the conventional markers alone or in combination with the methylomic markers, and the seven CpG sites in the final Cox model had a discriminative power of 89.9%. In this respect, the methylomic data also performed better than the transcriptomic mortality predictors in our previous study [10]. Nevertheless, we acknowledge that the major weaknesses of our study are a lack of a separate verification cohort and a rather small study population. Hence, the results must be considered as tentative and hypothesis-generating. The strength of our study, however, is the fact that all the study participants were 90 years of age at baseline. Therefore our results are not confounded by the effect of chronological age on DNAm.

A recent study by Moore et al. analyzed genome-wide methylomic mortality predictors in individuals with a wide age range (30-100 years at 9-year follow-up, mean mortality follow-up time 4.4 years) [23]. They identified 76 CpG sites where the rate of change in DNAm was associated with mortality and 88 markers where the year 9 level of DNAm was associated with mortality. Interestingly, their mortality-associated DNAm sites also included genes with immunoinflammatory functions and a link to NF-κB regulation. However, no overlap between individual mortality-associated CpG sites were found in our data sets. These differences may arise due to different population characteristics, such as age range and the causes of death. Hence, further studies are required to establish the potentially age and population-specific relationships between DNAm and mortality.

When we examined the seven final signature mortality-predicting CpG sites and their corresponding genes (Table 3) for overlap with the genes harboring the most commonly aging-associated CpG sites - *ELOVL2, FHL2* [2, 21, 24-26], *KLF14* [2, 21, 25, 26], *SST* [2, 25, 26], *OTUD7A* [2, 24, 26], *PENK* [2, 21, 24, 25] and *EDARADD* [2, 6, 24, 26] - we found no overlap between these markers. Moreover, none of our top 250 mortality-associated methylomic sites (Supplementary Table 2) were among the 525 common age-associated CpG sites that have been observed in more than one study (summarized in [21]). Moore et al. [23] have also in observed a similar phenomenon in their population: very few (< 0,05%) of the aging-associated CpG sits were also mortality-associated. These observations suggest that aging-associated epigenetic drift and the epigenetic control of the life span in old age might operate through different genomic mechanisms. This hypothesis is also in line with our previous findings on age-associated transcripts [27], which displayed very little similarity with mortality-predicting transcripts [10].

Despite the increasing body of data that suggests that several manifestations of organismal aging and development are of epigenetic origin, the associations reported thus far on DNAm and aging-phenotypes are scarce and/or the findings have been negative. Bell et al. (2012) examined the genome-wide associations between DNAm and 16 age-related phenotypes and found that two phenotypes - lung function and low-density lipoprotein levels - exhibited an association with one CpG site (cg16463460 in *WT1* and cg03001305 in *STAT5A*, respectively) and maternal longevity exhibited an association with two CpG sites (cg13870866 in *TBX20* and cg09259772 in *ARL4A*) [8]. In another EWAS, Marioni et al. (2015) detected no individual CpG sites associated with physical or cognitive fitness in an elderly population [28]. However, they did find a cross-sectional association between a measure of DNAm age - the epigenetic clock based on the Horvath predictor [17] -, and physical and cognitive fitness yet the DNAm age was not predictive of a longitudinal chance in the fitness measures [28]. The DNAm age has also been recently demonstrated to predict all-cause mortality in four different cohorts of elderly individuals [29] and in Danish twins [30]. However, the DNAm age was not predictive of mortality in our study. One reason for the negative finding might be that individuals in our cohort were all very old at baseline (90 years), and death at this age likely has different underpinnings than at younger old ages and when assessed in cohorts with wider age spectra.

In conclusion, the results of this study support the genomic-level role of NF-κB at the very end of the human life span. We hypothesize that our findings could relate to the recent observation of a programmatic role of hypothalamic NF-κB and IκB kinase-β activation in the control of the life span in experimental mouse models [16]. Adhering to the conclusion of this mouse study that the decisive role of hypothalamic NF-κB is exerted systemically level through immune-neuroendocrine crosstalk [16], we suggest that our findings on immune cells might represent the peripheral correspondence of hypothalamic NF-κB activation. However, establishing the systemic-level events that connect NF-κB function to all cause-mortality in aged humans will require further research.

## MATERIALS AND METHODS

### Study population

The study population consisted of nonagenarian subjects participating to the Vitality 90+ study, which is an ongoing, prospective population-based study on individuals aged 90 years and above and who reside in the city of Tampere, Finland. The Vitality 90+ study was initiated in 1995, and since then several nonagenarian cohorts have been recruited and examined for biological, clinical, demographic and social measures. Mortality rates have been analyzed longitudinally using complete follow-ups. The recruitment protocol and characterization of the subjects in the current study has been previously described [10]. The data in this study concern individuals born in 1920 and recruited in 2010 for sample collection. Genome-wide methylation data and the full covariate data including cell type proportions were available for 111 subjects (*n* = 81 women and *n* = 30 men). The all-cause mortality data were collected from the Population Register Center. As we wanted to assess both shorter and longer-term survival predictors for this cohort, the mortality data was collected in two different time points. The first data collection was performed on 31^st^ of January in 2013 corresponding to a 2.55-year median follow-up and the second one was on 31^st^ of May in 2014 corresponding to a 4-year follow-up. The mortality rate at the 2.55-year follow-up was 32.4% (36/111) and 47.7% (53/111) at the 4-year follow-up. All the participants gave their written informed consent. The study was conducted following the guidelines of the Declaration of Helsinki, and the study protocol was approved by the ethics committee of the city of Tampere.

### Sample collection and processing

Venous blood samples were collected in EDTA-containing tubes by a trained home-visiting medical student between 8 am and 12 am. Plasma was separated and stored at −70°C. Genomic DNA and total RNA were extracted from PBMCs in which the blood samples were subjected to leucocyte separation using a Ficoll-Paque density gradient (Ficoll-Paque™ Premium, cat. no. 17-5442-03, GE Healthcare Bio-Sciences AB, Uppsala, Sweden). The PBMC layer was collected, and the cells allocated for RNA extraction were suspended in 150 μl of RNAlater solution (Ambion Inc., Austin, TX, USA). Cells that were allocated to FACS analysis and DNA extraction were suspended in 1 ml of a freezing solution (5/8 FBS, 2/8 RPMI-160 medium, 1/8 DMSO; FBS cat. no. F7524, Sigma-Aldrich, MO, USA; RPMI: cat. no. R0883, Sigma-Aldrich, MO, USA; DMSO: cat. no. 1.02931.0500, VWR, Espoo, Finland).

Characterization of the subjects for their anthropometric measures, functional performance, plasma biomarkers and blood cell distributions (all parameters presented in Table 1) has been previously described (please see [10] and the references therein). In addition to these variables, in the current study we also determined a measure of the “epigenetic clock” - the DNAm age in the PBMCs - using the methodology presented in the study by Horvath et al. (2013) [17], (algorithm available athttps://dnamage.genetics.ucla.edu/). Initially, the age predictor was generated by Horvath with elastic net regression using *21,369* probes that are present in HumanMethylation450 as well as in HumanMethylation27 BeadChips. The predictor was trained with 8,000 samples of various tissue types in 82 Illumina DNA methylation array data sets. Based on the training results, the “epigenetic clock” i.e. the regression model was built with 353 CpG-sites whose methylation level explains most of the age variation.

### Illumina methylation array and preprocessing of the data

Genome-wide DNA methylation profiling was performed using the Infinium HumanMethylation450 BeadChip (Illumina, San Diego, CA, USA) according to the manufacturer's protocol at the Institute for Molecular Medicine Finland (FIMM) Technology Centre of the University of Helsinki. For bisulfite conversion, 1 μg of DNA was used (EZ-96 DNA Methylation Kit, Zymo Research, Irvine, CA, USA) and 4 μl of the bisulfite-converted DNA was subjected to whole-genome amplification and enzymatic fragmentation. Hybridization was carried out according to the manufacturer's protocol. Samples were run on the arrays in a randomized order and the chips were scanned with the iScan reader (Illumina).

The *methylumiset* object in the R software with the *wateRmelon* array-specific package from Bioconductor was used in preprocessing of the data. Probes mapping to sex chromosomes (n = 11,648) were also removed. In addition, all polymorphic sites and sites exhibiting unspecific probe binding (*n* = 76,775) were filtered out based on database information [31]. CpG target sites demonstrating technically poor quality were filtered out, including sites with a beadcount of < 3 in 5% of the samples (*n* = 515) and sites for which 1% of the samples had a detection *p*-value > 0.05 (*n* = 698). The annotation information for the CpG sites was retrieved using the GRCh37/hg19 genome assembly (released in February 2009). The *dasen* method was used for background correction and quantile normalization individually for the two applied chemistries in the Illumina platform (Infinium I and II) and for the intensities of methylation (m) and un-methylation (u). Following the *dasen* method, the u and m intensities were transformed to beta (β) and M values, where β is the ratio of the methylated probe (m) intensity in relation to the overall intensities (m + u + α), where α is the constant offset, i.e., 100. Lastly, the batch effect of the different chemistries was corrected using the BMIQ method, which is based on beta mixture models and the EM algorithm [32]. The batch effect produced by two different run series was corrected using an Empirical Bayes-based algorithm implemented in the R package *Combat.* Because the proportions of the CD4+CD28-, CD8+CD28− and CD14+ cells and the CD4+ to CD8+ cell ratio were associated with the variation in methylation data in the principal component analysis [33], the data was regressed in *the variable dispersion beta regression* model from Ferrari and Cribari-Neto [34] with the explanatory variables of gender and the proportions of blood cell types after which the standardized weighted residuals were extracted and used in all further statistical analyses. The model utilizes beta density function with parameterizations:

$$\varphi \left(y,\mu ,\phi \right)=\frac{\Gamma \left(\phi \right)}{\Gamma \left(\phi \right)\Gamma \left(1-\mu \right)}y^{\phi -1}{\left(1-y\right)}^{\left(1-\mu \right)\phi -1}$$,

where Γ(.) is the gamma function; *y* is the continuous response variable with a mean value of *μ*, which is assumed to follow a beta distribution inside the interval *y* ϵ (0,1); and ϕ < 0 is the precision parameter. The variance of *y* is inherited from the binomial variance *μ(1-μ)*, and it can be written as *μ(1-μ)/(1+ ϕ)*. Beta regression utilizes maximum likelihood for estimating the parameters in the equation, and the mean value of *y* is connected to the linear equation with the canonical link function *logit*. The model is implemented in the R package *betareg* as a default setting. The methylation data are available in the GEO database (http://www.ncbi.nlm.nih.gov/geo/) under the accession number GSE68194.

All the CpG sites passing the quality control and preprocessing criteria described above as well as the conventional variables presented in Table 1 were first analyzed for their univariate association with mortality after which all the significant methylomic markers (*p* < 0.05) were corrected for FDR with the B-H -method (FDR < 0.5). The Cox regression models were performed using Stata software (version 13.0 for Windows, StataCorp LP, TX, USA), and the corrections for FDR were performed using R version 3.0.2.

### Ridge regression

Due to the high dimensionality and multicollinearity of the genome-wide data, the standard Cox regression method cannot be directly applied to yield parameter estimates. Hence, several different dimension reduction and feature selection procedures have been presented for such data. In this study, we made use of the Ridge regression [35] that is based on penalized partial likelihood, and provides a means to avoid overfitting and unstable predictors. It has also been shown to produce reproducible results in whole-genome data sets by others [36] and us [10].

Ridge regression is a technique to analyze data when predictors are correlated with other predictors. In the presence of this multicollinearity, the variance of the regression coefficients is increased making them unstable. By adding a little bias (tuning parameter λ) to the coefficients, the Ridge regression reduces the variance considerably. In the Ridge regression, the regression coefficients are regularized by imposing penalties on their size. Thus, the coefficients are shrunk toward zero and toward each other, and the tuning parameter λ controls for the amount of shrinkage. There is no definitive rule for choosing λ, but the objective is to produce only a small increase in the weighted sum of square errors [37]. To select an optimal value of λ, a *k*-fold cross-validation is often performed. For the Cox proportional hazards model, Verweij and van Houwelingen [38] introduced a cross-validated partial log-likelihood method. In *k*-fold cross-validation, the data set is split in *k* pieces, using *k* - 1 of those used to build the model and from thereon validating on the *k*th, and *via* cycling through this assessment, validating on each of the *k* pieces sequentially, and then averaging or summing the *k* different deviances [39].

We estimated the optimal value of λ by maximizing the 10-fold cross-validated log partial likelihood. The optimal λ was then used to obtain parameter estimates for the different models, i.e., the conventional markers (MMSE and BMI) alone, the methylomic markers (the 19 CpG sites with an FDR < 0.5) alone and in combination with the above-mentioned conventional markers, and the methylomic markers ordered according to their statistical significance (*p*-value) in the univariate selection. The R package penalized was used in this assessment.

### Assessment of the predictive accuracy of modeling (generalizability) through cross-validation

We sought the most accurate mortality prediction model by assessing the differences in the deviances through cross-validation. The tested data sets were the above-mentioned three models i.e., the conventional markers alone, Ridge regressions containing the methylomic markers alone (Ridge1) and combined with the conventional markers (Ridge2) and the methylomic markers assessed through univariate selection. The procedure was performed following the guidelines presented by Bovelstad et al. (2011) [40]. In specific, the study population was randomly split 50 times into training and test sets (74 and 37 individuals, respectively). The difference in deviance between the fitted model and the null model containing no covariates is given by

$$\hat{\delta }=-2\{l^{\left(\text{test}\right)}\left({\hat{\beta }}_{\text{train}}\right)-l^{\left(\text{test}\right)}\left(0\right)\},$$

where $l^{\left(\text{test}\right)}\left({\hat{\beta }}_{\text{train}}\right)$ and $l^{\left(\text{test}\right)}\left(0\right)$ are the Cox log partial likelihoods for the test data evaluated at ${\hat{\beta }}_{\text{train}}$ and **o**, respectively. A small value of $\hat{\delta }$ is indicative of good performance.

### Assessment of the final mortality-predicting signature

The final signature predictive of mortality in the population with methylation data available (*n* = 111) was assessed at the 2.55-year follow-up. The variables (19 CpG sites with an FDR-corrected *p*-value < 0.5) were collected from the model demonstrating the best accuracy of prediction (i.e., the Ridge regression containing only the methylation markers) and assessed in a stepwise Cox multivariate regression model. AIC was used to select the Cox regression model congaing the best set of predictors.

### Pathway analyses

IPA (QIAGEN Ingenuity Pathway Analysis (IPA^®^, QIAGEN Redwood City, www.qiagen.com/ingenuity) was used to identify canonical pathways and networks for the mortality-associated genes harboring the CpG sites (presented in Supplementary Table 2). If a CpG site was mapped to more than one gene, each of the genes were included in the network and pathway analyses. A description and principles of the pathway analysis have been previously provided in more detail [10]. B-H correction for FDR was used to assess the significance of the pathways; canonical pathways were considered significant at *p* < 0.05 (corresponding to a -log *p* < 1.3).

### Correlations between the methylomic markers and gene expression

The genome-wide gene expression analysis was performed using HumanHT-12 v4 Expression BeadChip (Cat no. BD-103-0204, Illumina Inc., CA, USA) at the Core Facility of the Department of Biotechnology of the University of Tartu. Preprocessing and analysis of the data were performed as previously described [10]. Briefly, the lumi pipeline was used; the background was corrected with the bgAdjust.affy package, the data were log2-transformed and quantile-normalized. Poor-quality data and background noise were filtered out as follows: probes exhibiting expression levels of < 5 or > 100 in more than 5 (3.3%) samples per transcript were excluded. The gene expression data are available in the GEO database (http://www.ncbi.nlm.nih.gov/geo/) under the accession number GSE65218. The correlations between the transcript expression levels and CpG site methylation level (the standardized weighted residuals) were analyzed using Spearman's rho. In the analysis we included the top 19 mortality-associated GpC sites presented in Supplementary Table 2 and the corresponding transcripts with expression level above the selected threshold of 5 i.e., ATP5SL, FOXP1, HIVEP3, IQSEC1, ITPR3, MAP3K14, METAP1, RGS10, RIOK1 and VOPP1.

### Analysis of the mortality-associated CpG site loci for gene regulatory elements

To obtain further functional information about the mortality-predicting CpG sites, the single-base resolution locations of these sites were examined for gene regulatory elements using the Encyclopedia of DNA Elements (ENCODE) Consortium data [41] in the UCSC genome browser (http://genome.ucsc.edu/, accessed 02/2016). Specifically, we searched for TF binding sites (ChIP-seq data), genome states determined through combined genome segmentation data (ChromHMM and Segway programs) and DNAse I hypersensitivity clusters indicative of genomic regulatory regions. Default settings were used in inspecting the elements. However, we considered data only from cell types of blood origin; that is, the DNAse I hypersensitivity clusters and TFs were included in the results only if cells of blood origin were included in the cluster score, and for the analysis genomic states, data from GM12878 and K562 cells were accepted.

## SUPPLEMENTARY MATERIAL FIGURES AND TABLES






