# Supplementary material for: Methylomic predictors demonstrate the role of NF-κB in old-age mortality and are unrelated to the aging-associated epigenetic drift
Source: Oncotarget. 2016 Mar 22;7(15):19228–41. doi: 10.18632/oncotarget.8278 (PMC4991378; doi:10.18632/oncotarget.8278)
Supplement: Supplementary file 1 [file oncotarget-07-19228-s001.pdf]

Methylomic predictors demonstrate the role of NF-κB in old-age mortality and are unrelated to the aging-associated epigenetic drift

Supplementary Material

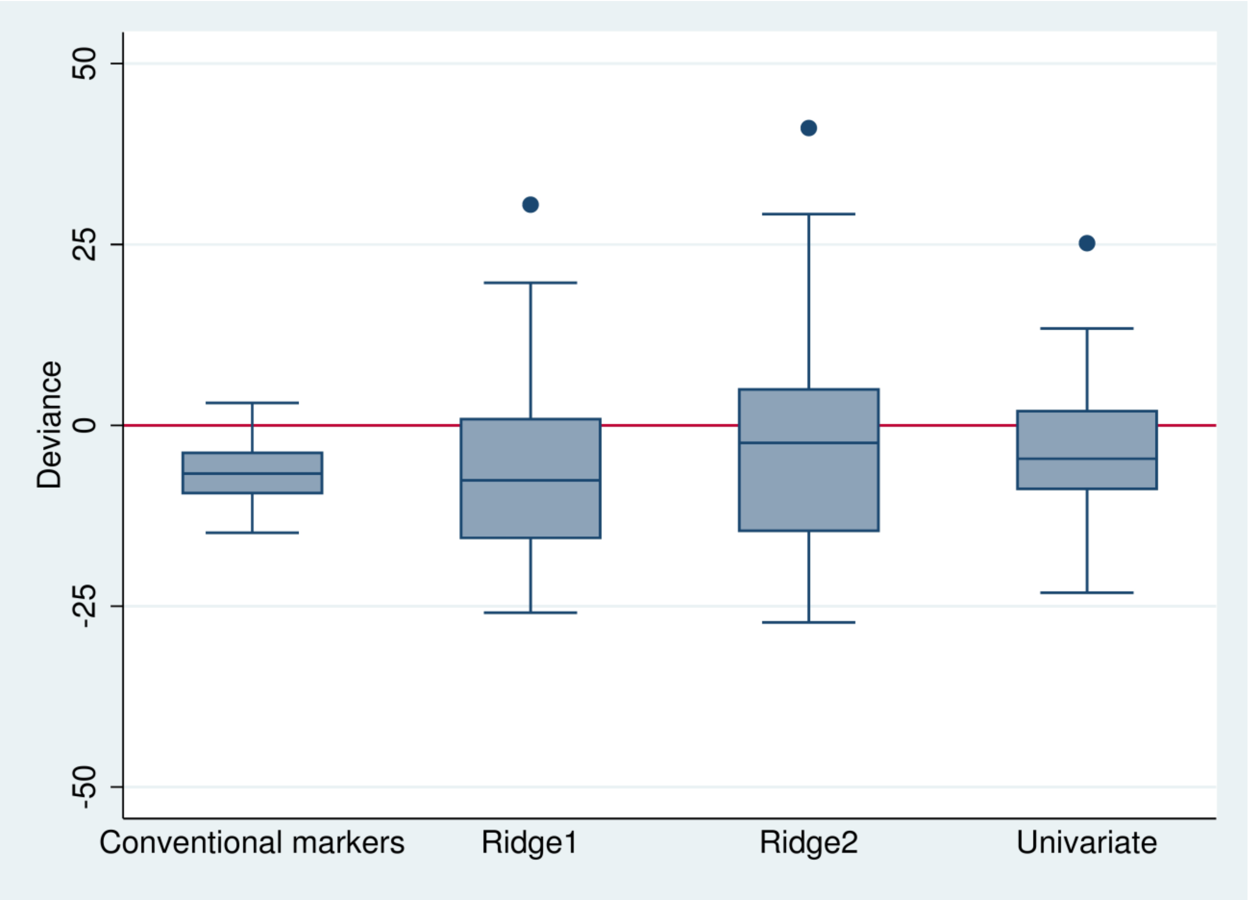

Supplementary Figure 1: Predictive accuracies based on the differences in deviance. The tested models were compared to the null model containing no covariates (the horizontal line at zero). The models contain the following variables: conventional markers alone, Ridge regressions containing the methylomic markers alone (Ridge1) and combined with the conventional markers (Ridge 2) and the methylomic markers based on univariate selection. A low value for the difference in deviance corresponds to a good predictive performance.

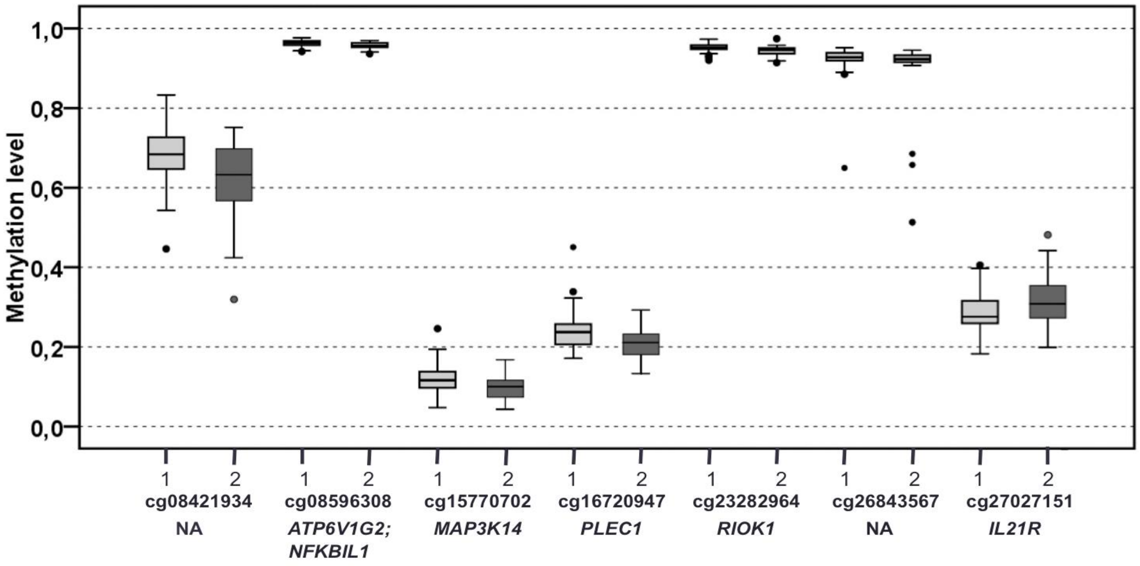

Supplementary Figure 2: Distribution of the beta values (DNA methylation level) of the mortality-predicting CpG sites according to the survival status in the 2.55-year follow-up; 1 corresponds to a survivor and 2 corresponds to a non-survivor.

Supplementary Table 1. The variables predicting 2.55-year mortality in the Cox univariate assessment are presented on the left side of the table and the variables remaining as independent predictors in the stepwise Cox multivariate model are presented on the right side of the table.

	**Univariate**		**Multivariate**	
	HR (95% CI)	p	HR (95% CI)	p
Hip circumference	0.94 (0.90-0.99)	0.009		
Waist circumference	0.97 (0.94-1.00)	0.035		
Weight	0.96 (0.93-0.98)	0.002		
BMI	0.86 (0.79-0.94)	0.001	0.88 (0.82-0.95)	<0.001
MMSE	0.91 (0.87-0.95)	<0.001	0.90 (0.86-0.95)	<0.001
Barthel index	0.98 (0.97-0.99)	<0.001		
Handgrip	0.95 (0.91-1.00)	0.032		
Not able to perform chair-rise test	2.26 (1.13-4.50)	0.021		
cf-DNA level	6.29 (1.56-25.35)	0.010		
Unmethylated cf-DNA level	6.24 (1.55-25.11)	0.010		
Frailty index (ref.=non-frail)				
Pre-frail	4.34 (1.30-14.52)	0.017		
Frail	6.46 (1.82-22.90)	0.004		
Frailty score	1.54 (1.16-2.04)	0.003		

Abbreviations: BMI, body mass index; cf-DNA, cell-free DNA; CI, confidence interval; HR, hazard ratio; MMSE, Mini-Mental State Examination

Supplementary Table 4. The Ridge regression -organized methylomic markers (FDR<0.5) in the 2.55-year follow-up data.

**Target ID**	**UCSC refgene name**	**Ridge regression coefficient**
cg08421934	NA	0.512181430
cg15770702	*MAP3K14*	0.440451714
cg08596308	*ATP6V1G2; NFKBIL1*	0.361064121
cg23282964	*RIOK1*	0.314444287
cg16720947	*PLEC1*	0.287521888
cg27027151	*IL21R*	0.282608719
cg02395768	*ATP5SL*	0.271127979
cg26843567	NA	0.235420720
cg24859528	*IQSEC1*	0.194700346
cg04395703	*METAP1*	0.162552256
cg03348466	*CRTC3*	0.155077082
cg03171419	*GPR124*	0.138358996
cg00291478	*RGS10*	0.103998955
cg22794214	*HIVEP3*	0.103551113
cg04182483	*RGS10*	0.070992030
cg08486432	*ITPR3*	0.065722362
cg08352439	*VOPP1*	0.063340550
cg21200667	NA	0.035298424
cg25356639	*FOXP1*	0.000682824

Supplementary Table 5. Spearman's correlations between 19 mortality-associated methylomic markers (FDR<0.5) and the corresponding gene product(s). Statistically significant correlations are shown in bold.

**CpG site (gene)**	**Transcript (nuID)**	**r**	**p**
cg02395768 (*ATP5SL*)	ATP5SL (HZdfbr376_595d6GWQ)	0.067	0.482
cg03348466 (*CRTC3*)	CRTC3 (NU1SQcNIfXlLtcFVKk)	**-0.238**	**0.012**
cg25356639 (*FOXP1*)	FOXP1 (iIM_jhP14TuoqIB1cE)	-0.045	0.636
cg25356639 (*FOXP1*)	FOXP1 (cY7t7uDf7f.6qKkipo)	-0.001	0.992
cg22794214 (*HIVEP3*)	HIVEP3 (EJF7X6oWN5buFKoIC8)	**0.231**	**0.015**
cg24859528 (*IQSEC1*)	IQSEC1 (fHtV.IjVF7SJRJ9qtc)	0.039	0.687
cg08486432 (*ITPR3*)	ITPR3 (oKnieZ_bp79OFn7.3U)	0.032	0.735
cg15770702 (*MAP3K14*)	MAP3K14 (TCDJFV9VPlUnyXfdUo)	-0.039	0.687
cg04395703 (*METAP1*)	METAP1 (lXSeVTNxvX9JL7ULKI)	0.144	0.131
cg00291478 (*RGS10*)	RGS10 (ljvQWZbiWniSgmllt)	-0.116	0.226
cg00291478 (*RGS10*)	RGS10 (fn1IPzBE4lVACWoekk)	-0.072	0.451
cg04182483 (*RGS10*)	RGS10 (ljvQWZbiWniSgmllt)	-0.163	0.088
cg04182483 (*RGS10*)	RGS10 (fn1IPzBE4lVACWoekk)	**-0.207**	**0.029**
cg23282964 (*RIOK1*)	RIOK1 (QRUS9J6e_P9HkHeX_4)	0.038	0.691
cg08352439 (*VOPP1*)	VOPP1 (93gq5.QBqkTenqC0To)	-0.080	0.406
